# Fibrinous pericarditis secondary to recurrent acute myeloid leukaemia

**DOI:** 10.1093/ehjcr/ytad537

**Published:** 2023-11-02

**Authors:** Takao Konishi, Taichi Kimura, Koichiro Minauchi, Shinya Tanaka

**Affiliations:** Department of Cardiovascular Medicine, Faculty of Medicine and Graduate School of Medicine, Hokkaido University, West 7, North 15, Kita-ku, Sapporo 060-8638, Japan; Department of Cancer Pathology, Faculty of Medicine, Hokkaido University, Sapporo, Japan; Department of Hematology, Sapporo City General Hospital, Sapporo, Japan; Department of Cancer Pathology, Faculty of Medicine, Hokkaido University, Sapporo, Japan; Institute for Chemical Reaction Design and Discovery (WPI-ICReDD), Hokkaido University, Sapporo, Japan

**Figure:**
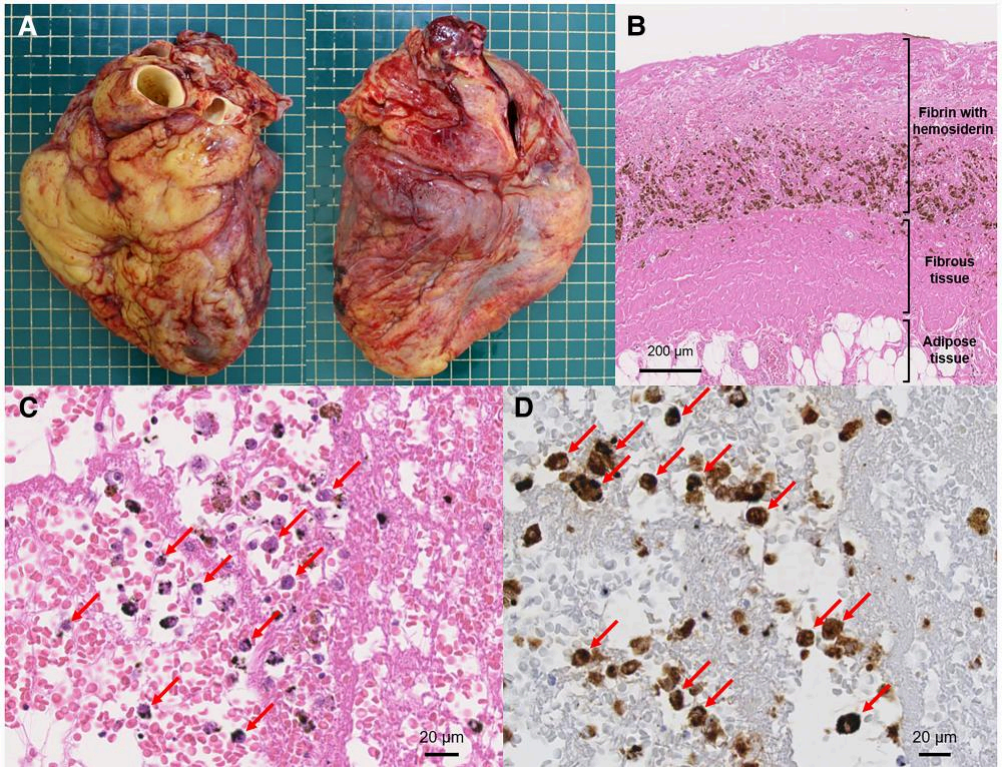


A 47-year-old man, who was diagnosed with acute myeloid leukaemia (AML), was hospitalized to receive remission induction therapy and haematopoietic stem cell transplantation. After the umbilical cord blood transplantation, pericardial effusion gradually increased despite a pericardiocentesis until pericardial fenestration was performed 5 months later. The patient later died of pneumonia and pulmonary haemorrhage. Autopsy revealed cardiomegaly (heart weight 590 g) with a 200 mL sanguineous pericardial effusion and a thickened epicardium with villus-like fibrinous adhesions on its surface (*Panel A*). Histological examination revealed an epicardial thickening of 8–10 mm due to the proliferation of organized fibrin, fibrous tissue and adipose tissue (*Panel B*). A high-power histological image demonstrated the infiltration of atypical inflammatory cells in the epicardium (*Panel C*). Immunohistochemistry for the epicardium showed that they were positive for myeloperoxidase (*Panel D*), suggesting the leukemic infiltration of the epicardium. The autopsy could identify the recurrence of AML as the aetiology of the pericardial effusion although the cytological examination of pericardial effusion derived from pericardiocentesis before his death was negative for malignancy. Cytologic examination was reported to be useful in the diagnosis of malignancy and exhibited a lower false-negative rate, compared to pericardial biopsy. This case suggests that, even if the pericardial cytology is negative for malignancy, clinicians should recognize the possibility of recurrent AML when a significant increase of pericardial effusion is observed after remission induction therapy or blood transplantation.

**Consent:** In this report, because this is an autopsy case report and patient information was entirely anonymized and de-identified, written informed consent was not obtained from the patient.

**Funding:** This work was supported in part by the research grant from The Ito Foundation (The 27th Ito Foundation Grant, TK).

**Data availability:** The data included in this article will be shared upon reasonable request to the corresponding author.

